# Zebrafish xenograft model of human lung cancer for studying the function of LINC00152 in cell proliferation and invasion

**DOI:** 10.1186/s12935-020-01460-z

**Published:** 2020-08-06

**Authors:** Wenyi Shen, Juan Pu, Jing Sun, Bing Tan, Wei Wang, Lili Wang, Jianmeng Cheng, Yangsong Zuo

**Affiliations:** 1 Department of Respiratory Medicine, Lianshui County People’s Hospital, Kangda College of Nanjing Medical University, Huai’an, 223400 China; 2 Department of Radiotherapy, Lianshui County People’s Hospital, Kangda College of Nanjing Medical University, Huai’an, 223400 China; 3 Department of Clinical Laboratory, Lianshui County People’s Hospital, Kangda College of Nanjing Medical University, Huai’an, 223400 China

**Keywords:** LINC00152, Zebrafish xenograft, Afatinib, EGFR, Lung cancer

## Abstract

**Background:**

Numerous studies have shown that long noncoding RNAs play important roles in human cancer progression. Although zebrafish xenografts have recently become a novel in vivo model for human cancer research, whether such models can be used to study the function of long noncoding RNAs remains unknown.

**Methods:**

In vitro studies validated the roles of LINC00152 in the proliferation and invasion of lung cancer cells. In vivo studies of zebrafish xenografts also confirmed these roles of LINC00152. In vivo confocal imaging was used to more accurately evaluate the function of LINC00152 in cell proliferation and migration. Pharmacological experiments were further performed to study the potential ability of LINC00152 downregulation combined with an EGFR inhibitor to treat tumors in cultured cells and the zebrafish xenograft model.

**Results:**

Silencing of LINC00152 suppressed cell proliferation and invasion in SPCA1 and A549 lung cancer cell lines in vitro. In the zebrafish xenograft model, knockdown of LINC00152 reduced the proliferation and migration of lung cancer cells, as indicated by the two imaging methods at different magnifications. Moreover, the knockdown of LINC00152 enhanced the inhibition effect of afatinib for lung cancer progression in cultured cells and the zebrafish xenograft model.

**Conclusion:**

Our study reveals the oncogenic roles and potential for LINC00152 to be a target for tumor treatment in lung cancer using zebrafish xenograft models, and the findings suggest that this model could be used for functional and application studies of human long noncoding RNAs in tumor biology.

## Background

Zebrafish xenografts have become an increasingly popular animal model for human cancer research in the last decade [1]. Thus far, different types of zebrafish xenografts have been established, such as melanoma, colorectal cancer, and breast cancer xenografts [1–5], and zebrafish patient derived xenograft (zPDX) models have also been generated by transplanting hundreds of cells from human tumor tissue with a high success rate [3, 5–8]. The relative stemness and immature immune environment of zebrafish larvae represent proper conditions for the growth of human cancer cells [9]. Compared with the mouse xenograft model, the transparent zebrafish larvae xenograft model offers the speed and intuitiveness for studies on the cellular resolution level with relatively large samples [1, 7, 8], which would contribute to the study of tumor biology. Thus, developing reliable zebrafish xenografts has become increasingly important for tumor research and personalized clinical testing.

Bioinformatics analyses with whole-genome sequencing data show that protein-coding sequences account for less than 3% of the human genome and most of the transcripts are noncoding RNA, such as tRNA, rRNA, miRNA and long noncoding RNA (lncRNA) [10]. lncRNAs are a class of transcripts longer than 200 nucleotides with limited protein coding potential [11]. Recent studies showed that lncRNAs play critical roles in proliferation, differentiation, metastasis, metabolism, and apoptosis in cancer progression by regulating the expression of protein-coding genes at the transcriptional, posttranscriptional and epigenetic levels [12–16]. Thus far, only lncRNA THOR has been reported to have an oncogenic role in tumorigenesis via a zebrafish model [17]. However, the homologies of most human lncRNAs cannot be found in zebrafish, which limits the application of zebrafish genetic models for tumor-related lncRNA research [18]. Zebrafish xenograft models offer the in vivo conditions of human cancer cells and do not have such restrictions, which prompts us to explore whether such models can be used to study the roles of human lncRNAs in cancer.

Long intergenic noncoding RNA 00152 (LINC00152) was first reported to be highly expressed in human gastric cancer tissues and cells [19], and it is located on chromosome 2p11.2 and has a transcript length of 828 nucleotides. Further studies also found that LINC00152 was overexpressed in gallbladder cancer, clear cell renal cell carcinoma, glioma, lung adenocarcinoma, etc., and it is involved in cell proliferation, invasion and apoptosis [20–25]. These studies showed that LINC00152 regulates tumor progression via signaling pathways, which indicates that LINC00152 might have a common oncogenic role in different human cancers [20, 23, 26–30]. In lung cancer, LINC00152 is highly expressed at ~ 2.63-fold, and its expression is associated with poor prognosis for lung adenocarcinoma patients [23, 31]. Considering its importance, we have attempted to validate its roles by using zebrafish lung cancer xenograft models.

In this study, we first verified the function of LINC00152 in cell proliferation and invasion in two lung cancer cell lines. Then, zebrafish xenografts are evaluated via stereomicroscopy to validate the oncogenic role of LINC00152 in vivo. By confocal imaging experiments, we more precisely confirmed LINC00152’s function. Furthermore, we studied the antitumor effect of the combination of LINC00152 knockdown and EGFR inhibition and found that LINC00152 and EGFR had synergetic effects for lung cancer treatment.

## Materials and methods

### Zebrafish

Adult zebrafish were maintained in a fish auto culture system (Haishen, China) at 28 °C under a 14–10 h light–dark cycle. Embryos were raised in 10% Hank’s solution that consisted of (in mM) 140 NaCl, 5.4 KCl, 0.25 Na_2_HPO_4_, 0.44 KH_2_PO_4_, 1.3 CaCl_2_, 1.0 MgSO_4_ and 4.2 NaHCO_3_ (pH 7.2). The zebrafish AB wildtype and transgenic line Tg(fli1a:EGFP) were used in our study [32]. Zebrafish handling procedures were approved by Nanjing Medical University.

### Cell culture

The human nonsmall cell lung cancer cell lines A549, H1299, H1975, SPCA1, PC9 and human bronchial epithelial cells 16HBE were obtained from Institute of Biochemistry and Cell Biology of Chinese Academy of Sciences (Shanghai, China). A549, H1975 and 16HBE cells were cultured in 1640 medium; and H1299, SPCA1 and PC9 cells were cultured in DMEM. Both media were supplemented with 10% FBS (Sciencell, USA), 100 µ/ml penicillin, and 100 µ/ml streptomycin, and then cultured in a humidified air atmosphere containing 5% CO_2_ at 37 °C.

### RNA extraction and qRT-PCR

Total RNA was extracted from cell lines using TRIzol reagent. Total RNA was reverse transcribed to cDNA using 1st Strand cDNA Synthesis SuperMix for qPCR kit (Takara, Dalian, China). We performed quantitative reverse transcription-PCR (qRT-PCR) analyses using SYBR Green Master Mix kit (Takara) following the manufacturer’s instructions to detect the expression levels of LINC00152 in different lung cancer cell lines. Data were analyzed based on the 2^−∆∆CT^ method, and glyceraldehyde 3-phosphate dehydrogenase (GAPDH) was used as the internal control. The primer sequences of LINC00152 were as follows: forward 5′-CACTGAAAATCACGACTCC-3′ and reverse 5′-AAATGGGAAACCGACCAGAC-3′. The primer sequences for GAPDH were as follows: forward 5′-GGGAGCCAAAAGGGTCAT-3′ and reverse 5′-GAGTCCTTCCACGATACCAA-3′.

### RNA interference

LINC00152 small interfering RNA (siRNA) and negative control siRNA were purchased from General Biosystems (China). A549 and SPCA1 cell lines were cultured in six-well plates, and then specific siRNAs were transfected into A549 and SPCA1 cell lines after 24 h using Lipofectamine 3000 reagents (Invitrogen, USA). The LINC00152 siRNA sequence is 5′-TGATCGAATATGACAGACACCGAAA-3′, and the negative control siRNA sequences are 5′-TTCTCCGAACGTGTCACGT-3′. Cells were harvested after transfection for 24 h, and knockdown efficiency was then quantified by qRT-PCR.

### Cell proliferation assay

Cell proliferation assay were performed using a Cell Counting Kit-8 (CCK-8, DOJINDO, Japan). LINC00152 siRNA and negative control siRNA were transfected into A549 and SPCA1 cell lines, which were then seeded in 96-well plates after transfection for 24 h. A total of 2 × 10^3^ cells were seeded in each well of 96-well plates. Cell proliferation was monitored on a microplate reader (BioTek Elx800, USA) by measuring the optical density at 450 nm every 24 h from 0 to 72 h according to the manufacturer’s instructions, and then data of each group were analyzed.

### Transwell assays

A549 and SPCA1 were transfected with LINC00152 siRNA and negative control siRNA. After 24 h of transfection, transfected cells were seeded in 24-well plates with 8-mm-pore size chamber inserts. Then, 4–5 × 10^4^ cells were plated into the upper chambers, which were diluted with serum-free culture medium. The upper chambers were then placed into the lower chambers of 24-well plates that included 800 μl medium with 10% FBS. After 24 h, the cells migrated to the bottom surface of the membrane, and then the cells were fixed with methanol and stained with 0.1% crystal violet for 30 min. The images were collected under an inverted microscope.

### Zebrafish xenograft injection

Cultured cancer cells were labeled with CM-DiI (Invitrogen, USA) before injection. Cultured cells were first collected and then washed three times with HBSS. Next, the cells were labeled with CM-DiI at 37 °C for 5 min, following by 15 min at 4 °C, and unincorporated dye was removed by rinsing three times with HBSS. The cells were then examined via fluorescence microscopy. Subsequently, 2-days-postfertilization (dpf) zebrafish larvae were mounted using 1.2% low-melting gel (Promega, USA), and then approximately 400 CM-DiI labeled cells were injected into the perivitelline space (PVS) of each larvae under a microinjector (Picosprizer III, USA). After injection, the xenografts were cultured at 34 °C. At 24 h postinjection (hpi), the zebrafish larvae with similar sizes of transplanted cells were collected for further analysis and then cultured at 34 °C until the end of experiments.

### In vivo imaging and quantitative analysis

At 4 days postinjection (dpi), the zebrafish larvae were also mounted using 1.2% low-melting gel for the imaging experiments. Imaging experiments were performed via stereomicroscope (MVX10, Olympus, Japan) or confocal microscope using a 20X water-immersion objective (Fluoview 1000, Olympus, Japan). The spatial resolution of the images was 1600 × 1200 (MVX10) or 1024 × 1024 pixels (Fluoview 1000).

### Drug administration

After 24-h transfection, the cells were seeded in 96-well plates and cultured in medium containing 2 nM afatinib (Selleck, China), and then cell proliferation was quantified by CCK-8. Transfected cells were also plated into the upper chambers, and the medium in the lower chambers contained 2 nM afatinib. Then, cell invasion was quantified. In the zebrafish xenografts, the 24-hpi zebrafish xenografts with a similar tumor size were randomly divided in two groups: Hank’s solution and Hank’s solution containing 1 nM afatinib. These larvae were raised for three consecutive days (3 days posttreatment, dpt) and then mounted for the imaging experiments.

### Statistics

Statistical analyses were performed using unpaired Student’s *t*-tests. *P* values less than 0.05 were considered to be statistically significant. All results are represented as the mean ± SEM.

## Results

### Knockdown of LINC00152 suppressed lung cancer cell proliferation and invasion in vitro

**Fig. 1:**
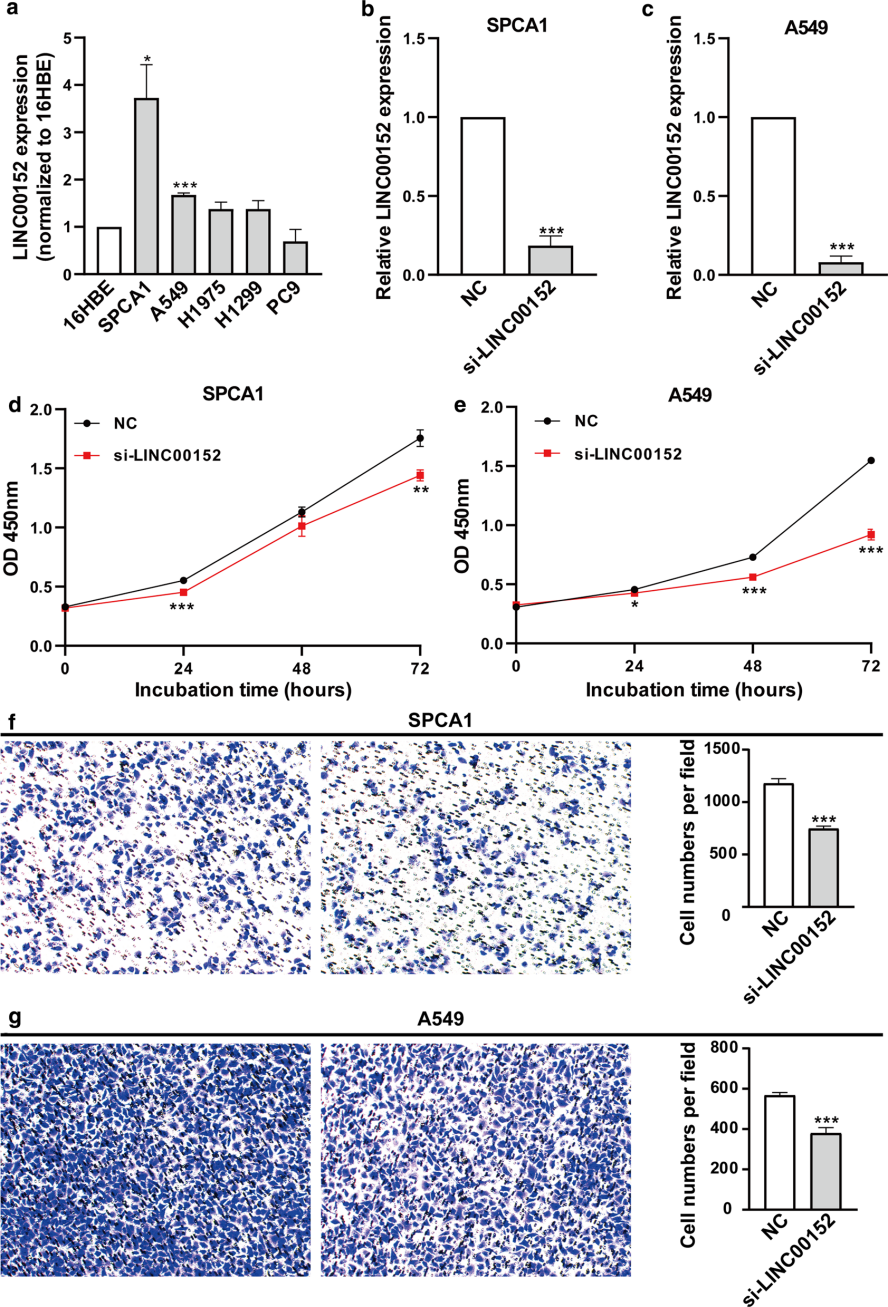
Knockdown of LINC00152 decreases the proliferation and invasion of lung cancer cells in vitro. **a** LINC00152 expression was analyzed by qRT-PCR in 5 NSCLC cell lines that were compared with the normal human bronchial epithelial cell line (16HBE). **b**, **c** LINC00152 mRNA levels in SPCA1 (**b**) and A549 (**c**) with LINC00152 siRNA (si-LINC00152) transfection compared with the negative control siRNA (NC) by qRT-PCR. **d**, **e** CCK-8 assays were performed to detect the viability of SPCA1 (**d**) and A549 (**e**) with LINC00152 siRNA transfection. **f**, **g** Transwell assays were performed to detect the invasion of SPCA1 (**f**) and A549 (**g**) with LINC00152 siRNA transfection. *: p < 0.05, **: p < 0.01, ***: p < 0.001

We first examined the LINC00152 expression level in five human lung cell lines using qRT-PCR via comparisons with the human bronchial epithelial cell line 16HBE. Among these cell lines, we found LINC00152 were highly expressed in the SPCA1 and A549 cell lines, and the values were 1.7 ~ 3.7-fold higher than that of the 16HBE line (Fig. 1a). To investigate the roles of LINC00152 in A549 and SPCA1, we tried to silence its expression in the two cell lines. According to the reported knockdown data of the three siRNAs [23], we synthesized one of the siRNAs with the highest silence efficiency and transfected it in SPCA1 and A549 cells. After 48 h posttransfection, we observed that the knockdown efficiency of LINC00152 was 81.5% in SPCA1 cells and 91.9% in A549 cells compared with the negative control siRNA (Fig. 1b, c). Next, we examined the cell viability using a CCK-8 assay and found that the viability of SPCA1 and A549 cells were significantly decreased when knocking down LINC00152 (Fig. 1d, e). Further, we examined cell invasion by transwell assays. We found that the knockdown of LINC00152 in SPCA1 and A549 cells also significantly repressed cell invasion compared with the control (Fig. 1f, g). These results verified the oncogenic roles of LINC00152 in the two lung cancer cell lines, suggesting that we could perform further in vivo tests in zebrafish xenografts using this siRNA-mediated knockdown strategy.

### Knockdown of LINC00152 suppressed lung cancer cell proliferation and invasion in the zebrafish xenograft model based on stereomicroscopy

**Fig. 2:**
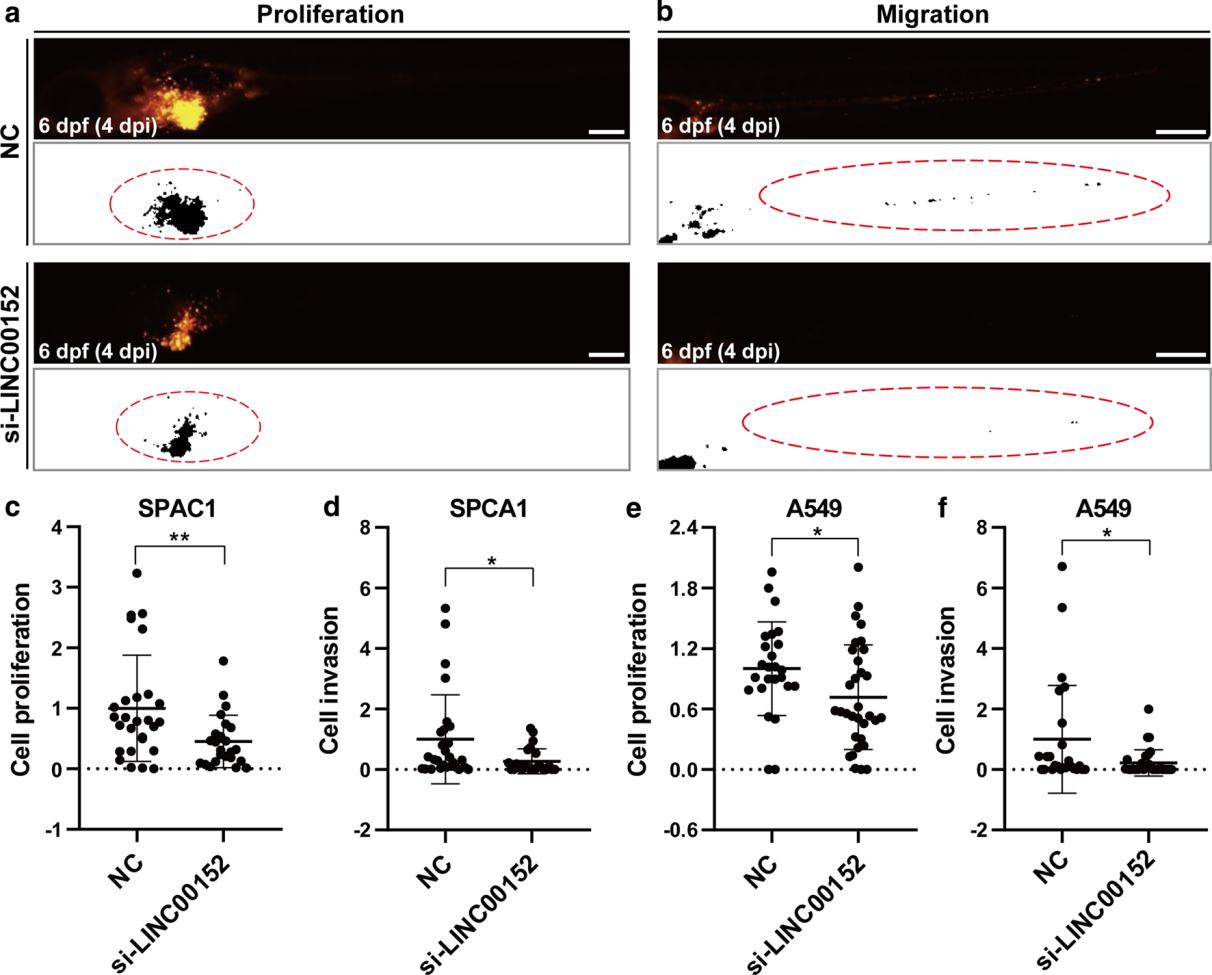
Knockdown of LINC00152 decreases the proliferation and invasion of lung cancer cells in zebrafish xenograft by stereomicroscopy. **a**, **b** Lung cancer cells transfected with si-LINC00152 siRNA or NC were injected into the PVS of 2-dpf WT zebrafish larvae. Images were taken using a stereomicroscope at 4 dpi. CM-DiI-positive areas in the yolk were quantified for proliferation (**a**), and CM-DiI-positive areas in trunk were quantified for invasion (**b**). The regions enclosed by the red dashed curve in the segmented images were selected for calculating tumor areas in the yolk or trunk. **c**, **d** Statistical analysis of proliferation (**c**) and invasion (**d**) when knocking down LINC00152 in SPCA1 cells. **e**, **f** Statistical analysis of proliferation (**c**) and invasion (**d**) when knocking down LINC00152 in A549 cells. *: p < 0.05, **: p < 0.01. Scale: 250 μm

To determine whether the knockdown of LINC00152 by siRNA affects tumorigenesis of lung cancer in zebrafish xenograft model, we transplanted the SPCA1 and A549 cells with LINC00152 siRNA transfection into zebrafish embryos. After 24 h posttransfection of siRNA, lung cancer cells were labeled with fluorescent dye (CM-DiI) and then approximately 400 cells were inoculated into the PVS of 2-dpf zebrafish larvae. To evaluate the tumor progression, we collected samples with similar tumor sizes via fluorescence stereomicroscopy from all the transplanted zebrafish larvae at 1 dpi, and these selected samples were then cultured. At 4 dpi, the yolk and trunk of the selected zebrafish larvae samples were photographed. We quantified the area with CM-DiI positive signals, which represented the tumor area in the yolk and trunk (Fig. 2a, b) [3, 33]. Compared with the control group, we found that the CM-DiI-positive area was significantly smaller in the yolk and trunk when knocking down the LINC00152 in SPCA1 cells (Fig. 2c, d), indicating that knockdown of LIN00152 decreased the proliferation (in yolk) and invasion (in trunk) of SPCA1 cells. Similar results were also observed in the A549 cells (Fig. 2e, f). These results are consistent with the data in cultured cells, indicating that the zebrafish xenograft model can be used for functional studies of LINC00152 in lung cancer by siRNA-mediated knockdown strategies.

### Knockdown of LINC00152 suppressed lung cancer cell proliferation and migration in the zebrafish xenograft model by confocal microscopy

**Fig. 3:**
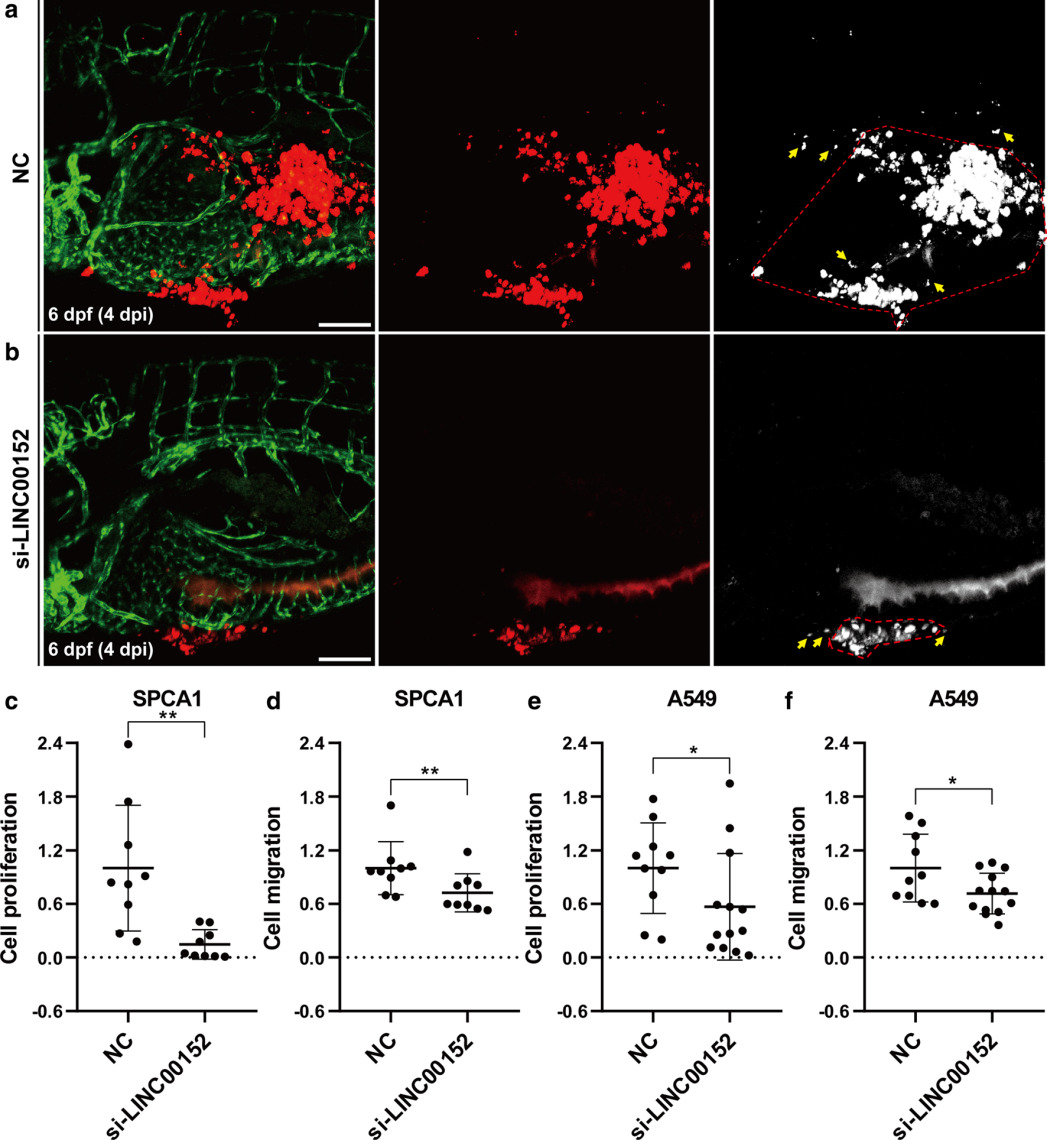
Knockdown LINC00152 decreases the proliferation and invasion of lung cancer cells in zebrafish xenograft by confocal microscopy. **a**, **b** Lung cancer cells transfected with si-LINC00152 (**a**) or NC (**b**) were injected into the PVS of 2-dpf Tg(fli1a:EGFP) transgenic zebrafish larvae. Images were taken by confocal microscope at 4 dpi. Tumor areas in the yolk were quantified for proliferation, and the polygons enclosed by red dashed lines represent the tumor cell covering areas. The yellow arrow represents cell debris, which is excluded from the tumor cell covering areas. **c**, **d** Statistical analysis of proliferation (**c**) and migration (**d**) when knocking down LINC00152 in SPCA1 cells. **e**, **f** Statistical analysis of proliferation (**e**) and migration (**f**) when knocking down LINC00152 in A549 cells.*: p < 0.05, **: p < 0.01. Scale: 100 μm

During the imaging experiments using the stereomicroscope, we found that the CM-DiI-positive signal might not always represent the tumor cells because nonspecific fluorescence signals were observed in the yolk of the zebrafish larvae. In addition, only a few of the transplanted SPCA1 or A549 cells were observed in the trunk of the larvae, which meant that the two cells had limited invasion levels in the zebrafish xenograft and additional samples must be analyzed to increase the credibility of the data. Thus, we used confocal microscope to obtain high-resolution images with few nonspecific signals and then analyzed the proliferation and migration in the yolk. To simultaneously examine the tumor progression and angiogenesis, CM-DiI labeled lung cancer cells were injected into Tg(fli1a:EGFP) zebrafish larvae in which the vascular endothelial cells are labeled by EGFP, and then the xenografts were imaged by multiple-layer scanning at 4 dpi (Fig. 3a, b). In these confocal images, we also found a few separate CM-DiI-positive spots that could not be identified as cancer cells because the size of spots was much smaller than the cells (right in Fig. 2a, b). We quantified the area of CM-DiI-positive cells by removing the noncell spots in the yolk and then comparing the LINC00152 siRNA transfected group with the NC transfected group in the SPCA1 cells. We found that the knockdown of LINC00152 decreased the cell proliferation significantly (Fig. 3c). To analyze the cell migration in the yolk, we first quantified the cancer cell coverage area by calculating the area of polygon formed by connecting lines of peripheral cancer cell points, which represented the total range of cell migration. To avoid the effects caused by the number of total cancer cells, we quantified cell migration by dividing the cell coverage area by the tumor area. We found that the knockdown of LINC00152 significantly decreased the migration of SPCA1 cells in the yolk of zebrafish larvae (Fig. 3d). Similar results were also observed in the A549-cell transplanted larvae (Fig. 3e, f). These results confirm that the zebrafish xenograft model can be used for functional studies of LINC00152 in lung cancer.

### Knockdown of LINC00152 and blocking of EGFR have a synergetic effect on the inhibition of proliferation and invasion of lung cancer in vitro

**Fig. 4:**
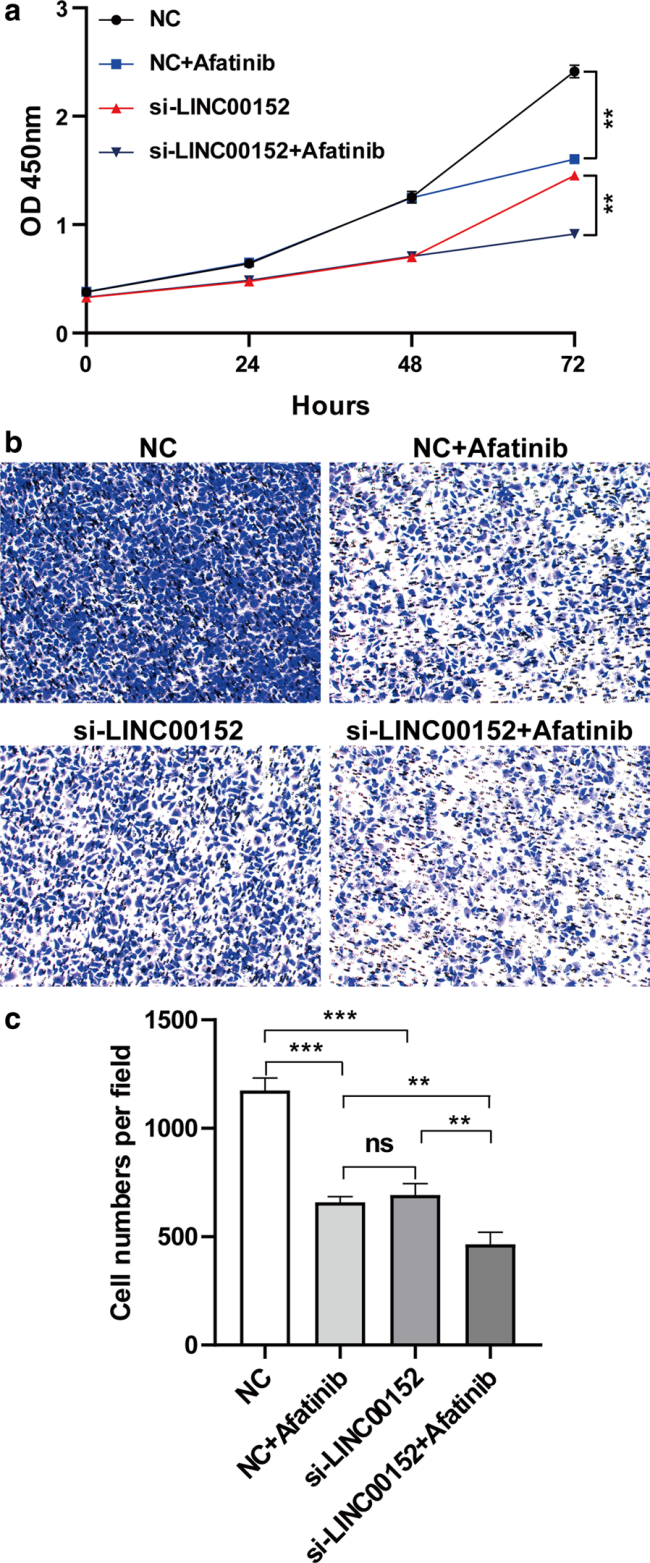
Knocking down LINC00152 and blocking EGFR have a synergic effect on the inhibition of cell proliferation and invasion in vitro. **a** CCK-8 assays were performed to detect the viability of A549 with NC transfection, NC transfection and afatinib (EGFR inhibitor) administration, si-LINC00152 transfection, si-LINC00152 transfection and afatinib administration. **b** Transwell assays were used to investigate invasion with NC or si-LINC00152 transfection and afatinib administration or not. **c** Statistical analysis of invasion with NC or LINC00152 siRNA transfection and afatinib administration or not. ns, not statistically significant, **: p < 0.01, ***: p < 0.001

Next, we tested the possibility of LINC00152 as a treatment target of lung cancer. EGFR is one of the critical therapeutic targets in NSCLC lung cancer, and the A549 cell line is sensitive to different types of EGFR inhibitors [34, 35]. The knockdown of LINC00152 has been reported to decrease EGFR expression, which indicates that LINC00152 might regulate cancer progression via the EGFR pathway [36]. Accordingly, we first examined the viability of A549 cells when blocking EGFR by afatinib and found that cell viability was significantly decreased, which was similar to the effect of knockdown of LINC00152 (Fig. 4a). We also noticed that knockdown of LINC00152 led to an earlier inhibition effect than afatinib treatment in A549 cells, which implied that LINC00152 could be upstream of the EGFR pathway. Moreover, simultaneous knockdown of LINC00152 and treatment with afatinib could enhance the inhibition effect (Fig. 4a). A transwell assay also found enhanced inhibition of invasion in A549 cells (Fig. 4b, c). These results imply silencing of LINC00152 and blocking of EGFR might have a synergetic effect in lung cancer progression.

### Knockdown of LINC00152 and blocking of EGFR have a synergetic effect on the inhibition of proliferation in zebrafish xenograft model

**Fig. 5:**
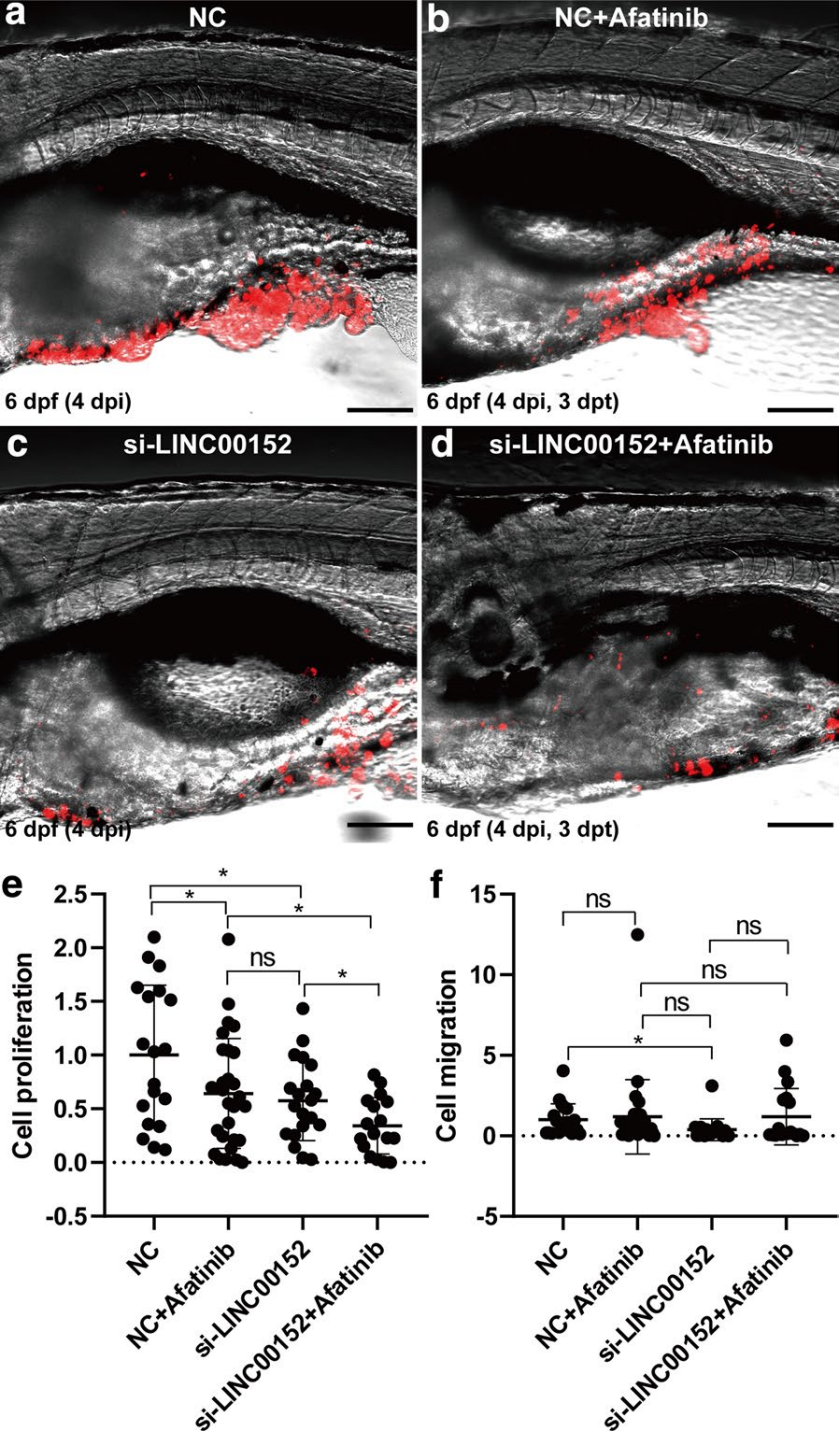
Knockdown of LINC00152 and block of EGFR have a synergic effect of inhibition in cell proliferation in zebrafish xenograft. **a**-**d** Lung cancer cells with NC transfection (**a**, **b**) or si-LINC00152 transfection were injected into the PVS of 2-dpf WT zebrafish larvae, and afatinib was bathed in zebrafish culture medium (**b**, **d**) or not (**a**, **c**). Images were taken by a confocal microscope at 3 dpt. **e**, **f** Statistical analysis of the proliferation and invasion with NC or LINC00152 siRNA transfection and afatinib administration or not. ns, not statistically significant, *: p < 0.05. Scale: 100 μm

We also tested the possibility of using LINC00152 for clinical application in zebrafish xenograft models. In addition to knockdown of LINC00152 in zebrafish xenografts, we also bathed afatinib in zebrafish culture medium (Fig. 5a–d). In the cultured cells, knockdown of LINC00152 also enhanced the inhibition effect of afatinib on cell proliferation in zebrafish lung cancer xenograft models (Fig. 5e). However, we did not observe enhanced inhibition of invasion when combining knockdown of LINC00152 and afatinib treatment (Fig. 5f). These results suggest that LINC00152 and EGFR might be clinical combined therapy targets for lung cancer.

## Discussion

Recent reports have indicated that LINC00152 plays important roles in the progression of lung cancer [23, 31, 37]. Further studies revealed that LINC00152 regulates the cell cycle of lung cancer through different signal pathways [23, 31]. In the present study, we verified that LINC00152 played an oncogenic role in cell proliferation and invasion in vitro. We also showed that knockdown of LINC00152 decreased the proliferation and migration of lung cancer cells in zebrafish xenograft models by two imaging experiments with different resolutions. Moreover, pharmacological experiments also indicated that LINC00152 and EGFR have synergistic effects on anti-tumor treatment in vitro and in vivo. These findings suggest that the zebrafish xenograft model could represent a new in vivo model for the functional study of human lncRNA.

Mouse xenografts are used for tumor biology for decades [38]. Previous studies also reported that LINC00152 promotes lung cancer proliferation and invasion in mouse xenograft models [23, 31]. At 18 days after subcutaneous injection in nude mice, A549 cells stably transfected with LINC00152 shRNA showed decreased tumor size and tumor weight, and immunohistochemical analyses showed a lower proliferation level in the LINC00152 silenced group than the control [23]. At 28 days after tail vein injection in nude mice, the metastatic cancer lesions in lung and liver tissue formed in the control mice but could not be detected in the LINC00152 silenced group [31]. Consistent with these results, our present study found that knockdown of LINC00152 decreased the proliferation and invasion of lung cancer cells in zebrafish xenograft models. These data suggest that zebrafish xenografts could be a reliable cancer model for human lncRNA functional studies.

Zebrafish xenografts not only offer an alternative strategy for in vivo studies but also show great promise in tumor biology. First, the zebrafish xenografts in the present study only could be evaluated 4 days after transplantation to determine the proliferation and invasion of tumor cells, whereas mouse xenografts require 2–4 weeks and stable shRNA transfection cells must be generated because the knockdown effect by siRNA transfection does not persist over such time periods. Second, different models are required to study proliferation and invasion in mouse xenografts, whereas different tumor cell distributions can be assessed throughout the body of zebrafish larvae using zebrafish xenografts; thus, proliferation and migration can be analyzed simultaneously. Additional parameters of tumor cells could be further analyzed, such as tissue-specific invasion and proliferation. Third, transparent zebrafish xenografts offer high-resolution cell observations, which means that fine tumor cell behavior can be detected in vivo. Combining transgenic lines that label different cell types, zebrafish xenografts offer good models for studying the relationship between tumor cells and different cells in vivo [2]. With the help of fast and high-resolution microscopy, timely and three-dimensional in vivo imaging has been achieved for observing the process of tumor cell metastasis in zebrafish larva [39]. In our study, we also evaluated vascular growth in the zebrafish lung cancer xenograft, although obvious changes in angiogenesis at the tumor sites were not observed, which indicated that the two lung cancer cell lines might not present obvious changes in vascular growth in our observation windows (Fig. 3a, b). Finally, the cost of zebrafish xenografts is much lower than that of mouse xenografts, which makes it possible to analyze larger samples for obtaining more accurate data for cancer research.

During the process of studying the zebrafish xenograft, we noticed that CM-DiI-positive signals do always represent tumor cells and may indicate the debris of cancer cells, such as apoptotic bodies, exosomes, etc., which would affect the accuracy when evaluating cell proliferation and invasion. In our study, a high-resolution imaging strategy can reduce the related impact, and we will attempt to improve it by florescent protein-expressing cell transplantation in further studies. Zebrafish larvae show a high success rate for generating xenograft models, even PDX models, although the relative stemness environment for cancer cells might be different from the conditions in human patients. Immune-deficient and transparent adult zebrafish models are also developed to generate xenograft models, which could improve the application of such xenografts in tumor biology [5]. In total, zebrafish xenografts offer rapid and intuitive models that represent a complementary and optimized model relative to mouse models. The development of the reliable analysis methods will expand the applications in basic and clinical research.

Drug testing using in vivo model is very critical for personalized therapy. Zebrafish xenografts can be proper models because easily drug administration and short evaluation time. In our study, pharmacological experiments indicated that LINC00152 might be a combination therapy target with EGFR for lung cancer. Moreover, a few of studies have been reported that zPDX model can be constructed that could be produced considerable amount of xenograft samples for personalized drug testing before clinical treatment [3, 5–8]. Although more studies are required for collecting the evidence of the consistency between human tumor and zPDX models, the zPDX model might represent a more feasible method than mouse models for personalized therapy.

## Conclusions

In summary, our results demonstrated the oncogenic effect of LINC00152 in zebrafish xenograft in vivo models. Additionally, pharmacological experiments indicated that knocking down LINC00152 and blocking EGFR have synergic effects on inhibiting lung cancer progression. Our study suggests that zebrafish xenografts represent a rapid and intuitive in vivo cancer model for lncRNA research.

## Data Availability

Not applicable.

## Abbreviations

CCK-8: Cell Counting Kit-8
dpf: Days postfertilization
dpi: Days postinjection
dpt: Days posttreatment
GAPDH: Glyceraldehyde 3-phosphate dehydrogenase
hpi: Hours postinjection
LINC00152: Long intergenic noncoding RNA 00152
lncRNA: Long noncoding RNA
PVS: Perivitelline space
qRT-PCR: Quantitative reverse transcription-PCR
siRNA: Small interfering RNA
zPDX: Zebrafish patient derived xenograft
